# Translumbar hemodialysis long-term catheters: an alternative for vascular access failure

**DOI:** 10.1590/2175-8239-JBN-2018-0080

**Published:** 2018-09-21

**Authors:** Fernando Moura, Felipe Leite Guedes, Yuri Dantas, Ana Helena Maia, Rodrigo Azevedo de Oliveira, Artur Quintiliano

**Affiliations:** 1 Universidade Federal do Rio Grande do Norte, Departamento de Radio-intervenção, Natal, RN, Brasil.; 2 Universidade Federal do Rio Grande do Norte, Divisão de Nefrologia, Natal, RN, Brasil.; 3 Universidade Federal do Rio Grande do Norte, Natal, RN, Brasil.

**Keywords:** Renal Dialysis, Vascular Access Device, Vascular Patency, Catheters, Indwelling, Treatment Outcome, Diálise Renal, Dispositivo de Acesso Vascular, Patência, Cateteres de Demora, Resultado do Tratamento

## Abstract

**Introduction:**

Vascular access (VA) in hemodialysis (HD) is essential to end-stage renal disease (ESRD) patients survival. Unfortunately, after some years in HD program, a significant number of patients may develop VA failure for many reasons. In this situation, arterial venous fistula (AVF) confection or catheters placement in traditional vascular sites (jugular, femoral or subclavian) are not feasible. In this scenario, translumbar tunneled dialysis catheter (TLDC) may be a salvage option.

**Objectives:**

To describe placement technic, complications, and patency of 12 TLDC.

**Methods:**

A retrospective study was performed to analyze 12 TLDC placement in an angiography suite using fluoroscopic guidance at the University Hospital of the Rio Grande do Norte Federal University from January 2016 to October 2017. The data collected of the total procedures performed consisted of demographic characteristics, success rates, observed complications, patient survival, and catheter patency.

**Results:**

All 12 TLDC were placed with success; there were only 2 significant periprocedure complications (major bleeding and extubation failure); 41.6% of patients presented a catheter-related first infection after 98 ± 72.1 (6-201) days, but catheter withdrawal was not necessary, mean total access patency was 315.5 (range 65 - 631) catheter-days, and catheter patency at 3, 6 and 12 months was 91 %, 75%, and 45%.

**Conclusion:**

TLDC is an option for patients with VA failure, improving survival and acting as a bridge for renal transplantation.

## Resumo

**Introdução:**

O acesso vascular (AV) para hemodiálise (HD) é crucial para os pacientes portadores de doença renal crônica (DRC) estágio V. Infelizmente, com o passar dos anos, um percentual não desprezível desses enfermos evolui para falência de AV por diversos motivos, o que impossibilita a confecção de novas fístulas arteriovenosas (FAV) ou o implante de cateteres venosos centrais nos sítios de punções tradicionais. Nesse cenário, o implante de cateteres translombares para hemodiálise (CTLHD) em veia cava inferior ganha destaque como medida salvadora.

**Objetivos:**

Relatar uma série de 12 casos de implante de CTLHD, sua técnica de implante, patência e complicações.

**Métodos:**

Estudo retrospectivo que analisou 12 implantes de CTLHD por radiologista intervencionista no setor de hemodinâmica do Hospital Universitário da Universidade Federal do Rio Grande do Norte (UFRN), no período de janeiro/2016 a outubro/2017. Os dados coletados consistiram em: características demográficas da população estudada, taxa de sucesso, complicações observadas, sobrevida dos pacientes, patência do cateter e desfechos clínicos.

**Resultados:**

Todos os 12 CTLHD foram implantados e utilizados com sucesso; ocorreram apenas 2 complicações associadas ao procedimento (sangramento e falha na extubação); 41,6% dos pacientes apresentaram infecção relacionada ao cateter após 98 ± 72,1 dias (6-201 dias), mas não houve necessidade de remoção; e a patência foi de 315,5 cateteres-dia (65-631 dias).

**Conclusão:**

O CTLHD é uma opção para pacientes com falência de acesso vascular, prolongando a sobrevida dos pacientes e atuando como ponte para o transplante renal.

## INTRODUCTION

In 2010, 2 million people worldwide received dialysis treatment to stay alive. About 5% of them are withdrawn from treatment, because of the lack of vascular access (VA) for hemodialysis (HD)^1^^,^ 2. Some of these patients had exhausted traditional vascular access to make an arteriovenous fistula (AVF) or to lodge a central venous catheters (CVC) or had *contraindication* for *peritoneal dialysis*. Potential alternative options such as extensive surgeries or CVC using unconventional accesses are associated with significant morbidity burden and have had limited success^3^. Transhepatic and translumbar inferior vena cava catheters have been used in these patients who have no other access site option. Translumbar tunneled dialysis catheter (TLDC) might offer a relatively safe and effective dialysis access option for patients with limited central venous access. However, additional studies are needed to estimate the long-term patency and safety of TLDC in this high-risk population^3^. We report our experience with the patency and complications of TLDC and patient survival at our institution.

## PATIENTS AND METHODS

We retrospectively reviewed the records of adult patients at the Division of Nephrology of Rio Grande do Norte Federal University who had received TLDCs between January 2016 to October 2017. All patients who had exhausted conventional access options, such as AVF and dialysis catheters for long-term dialysis, and had psycho-social contraindication for peritoneal dialysis were considered for inclusion in the study. Patient demographics, comorbid conditions, dialysis details (etiology of end-stage renal disease, HD length, catheter procedure, and previous access site), catheter insertion procedures and associated complications, catheter patency, and patient survival were retrieved from the medical records.

The study protocol was approved by the local Ethics Committee (CAAE 86448218.9.0000.5292).

### Techniques of catheter insertion

TLDCs were placed in an angiography suite using fluoroscopic guidance. Coagulation parameters (partial thromboplastin time, prothrombin time, platelet count) were checked and corrected before procedure as necessary. TLDC insertion was performed under moderate intravenous sedation and general anesthesia with 3-5 mcg/kg fentanyl, 2-3 mg/kg propofol, and 0.1 mg/kg cisatracurium. Prophylactic antibiotics were administered before catheter placement (1g cefazolin). Breathing, pulse, blood pressure, and oxygen saturation were monitored during all the procedure. All patients were informed about the procedure and informed consent was obtained. Procedure were performed by an experienced interventional radiologist. All patients in our sample used tip dual lumen polystyrene catheter (MEDCOMP Inc., Harleysville, PA, USA), 14.5 French diameter, with an overall and implant length cuff to tip of 52cm and 35cm, respectively.

**Figure 1:**
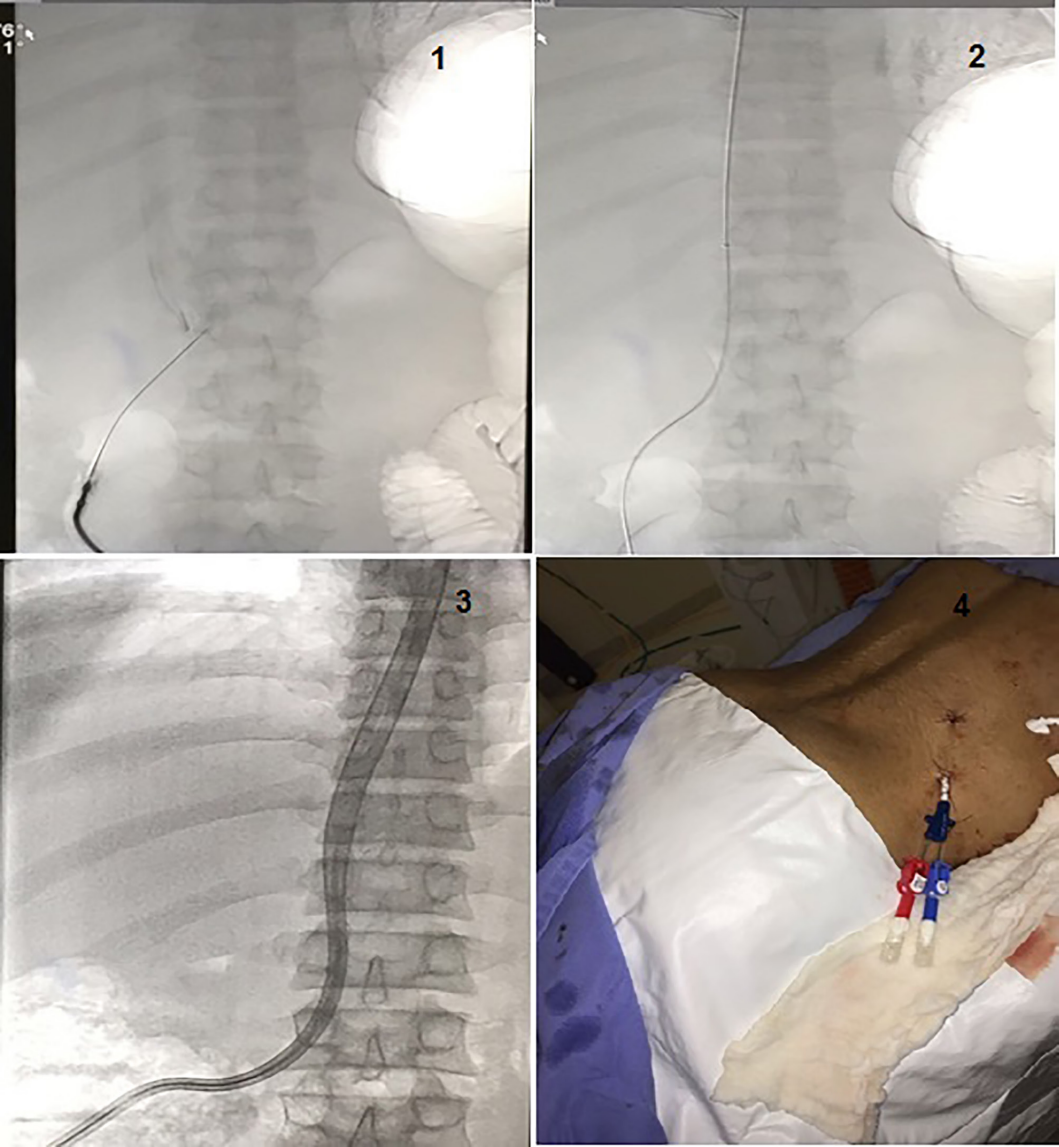
Translumbar catheter insertion procedure. 1) Trajectory and pathway of the access needle into the IVC 2) Guide-wire into the IVC 3) Dialysis catheter in the IVC 4)Catheter placed and the patient in prone position.

Routinely, a single access technique was used to perform the translumbar placement, as follows: The patient is placed in prone or prone oblique decubitus position and the skin prepared and draped from below the iliac crest to the lower ribs and from the spine to the mid abdomen. A small incision is made just above the right iliac crest at the L3 vertebral level. Through this incision, a 21-gauge, 15-cm-long needle is advanced under fluoroscopic guidance through subcutaneous tissues and back muscles (erector spinae and psoas) toward the inferior vena cava (IVC), below the renal veins. Once the translumbar needle enters the IVC, a guidewire is placed and the translumbar catheter is ultimately advanced until the tip is in the right atrium. Dacron cuff is tunneled under the skin and sutured. The translumbar exit site should be above the beltline and as far anterior as possible (eg, midaxillary line) for patient comfort. Heparin locks are used per institutional protocol. To prevent bleeding, if the HD will be perform in the first 24 hours after procedure, the heparin should be administered in lower doses (50 u/kg - 5000 u/mL). The use of contrast dye is planned, and the patient should be premedicated when a known contrast allergy exists. But the use of contrast dye is just to verify the catheter position. After placement of the TLDC, leg and back pain is expected for several days and can be managed with mild analgesics (Figure 1).

### Definitions

We used the definitions proposed by the Society of Interventional Radiology (SIR)^4^. According to those definitions, initial (primary) device service interval is defined as the number of catheter days from TLDC insertion until device failure or removal at the completion of therapy, patient death, or conclusion of the study with the catheter still functioning. Revised (secondary) device service interval is defined as the service interval that begins after device replacement or salvage, without abandonment of the access site. Device failure is defined as any limitation in catheter function, despite a technically successful placement. The target blood flow for CVCs is ≥300 mL/min.

### Complications

According to SIR^4^, all the complications, including pulmonary and cardiac events occurring within 30 days after the procedure, are considered related to the procedure. Minor complications are those that require no specific therapy and are resolved without any adverse consequence. The main complications are defined as those that require an increase in the level of care or result in hospitalization, permanent adverse sequelae, or death. If a complication cannot be successfully treated, it results in failure of the procedure. Late bleeding is defined as a bleeding episode requiring medical management after initial hemostasis had been achieved. Hypotension (SBP ≤ 90 mmHg or DBP ≤ 60 mmHg), oxygen saturation ≤ 90%, and extubation failure at any time during the procedure are considered as complications. Overall complications included all complications occurring at any time during the study follow up.

CVC dysfunction is defined as failure to attain a sufficient extracorporeal blood flow of >300 mL/min. Device failure can be due to multiple reasons, including mechanical reasons, such as kinking, retraction or dislodgment, thrombosis, formation of a fibrin sheath, infection, and poor blood flow. Catheter-related infection (CRI) include phlebitis, exit site infection, pocket infection, and catheter-related blood stream infection (CRBSI). Exit site infection is defined as the presence of new erythema, pain at the exit site and/or purulent drainage around the catheter. CRBSI is defined as a positive blood culture in a febrile patient and absence of clinical signs of a non-catheter-related source of infection^5^.

### Statistical analysis

Categorical variables are reported as frequency and percentage. Survival analysis was obtained using the Kaplan-Meier method. Statistical analysis was performed using IBM SPSS^®^ 22 Statistics.

## RESULTS

### Patient characteristics

There were 12 TLDCs (11 primary insertions and 1 exchange) inserted in 11 patients during the study period. Mean age of the study population was 56.7 ± 19.2 years with 50% being males. The major causes of ESRD were hypertension (41.6%) and diabetes mellitus (33.3%). The majority of patients were hypertensive (91.6%), diabetics (50%), and overweight or obese (50%). Mean time on dialysis at the beginning of the study was 63 ± 22.3 months. The number of previous AVF and catheters were 2.9 ± 2 and 10.3 ± 5.5, respectively.

More detailed information about demographics is available in Table 1

**Table 1:** General characteristics of the patients.

Variables	N = 12
Gender – male (n, %)	6 (50)
Age (years)	56.7 ± 19.2
Time on dialysis (months)	63 ± 22.3
Etiology of end-stage renal disease (n, %)	
Hypertension	5 (41.6)
Diabetes mellitus	4 (33.3)
Chronic glomerulonephritis	2 (16.7)
Post-renal kidney injury	1 (8.4)
Comorbidities (n, %)	
Hypertension	11 (91.6)
Diabetes mellitus	6 (50)
Overweight/obesity	6 (50)
Neoplasia	2 (16.7)
Congestive Heart failure	2 (16.7)
Dementia	2 (16.7)
Previous catheter (n)	10.3 ± 5.5
Previous AVF (n)	2.9 ± 2
Active waiting list candidates for kidney Tx (%)	41.6

AVF = arteriovenous fistula; Tx = transplantation

### Catheter outcomes

All TLDC insertions were successful, with good blood flows during the first session of dialysis (>300 mL/min) and two peri-procedural complications (one major bleeding and one extubation failure). A statistically significant difference (p < 0.05) in all clinical and laboratorial parameters before and after the TLDC placement - Table 2

**Table 2:** Assessment of clinical and laboratory parameters before and after TDLC placement.

Hemoglobin (pre/post – g / dL)	7.71 ± 0.91 / 9.83 ± 1.55	p < 0.001
Kt / V (pre/post) (m, sd)	0.32 ± 0.25 / 1.04 ± 0.16	p = 0.0002
Interdialytic weight gain (pre/post - L)	4.65 ± 0.82 / 2.5 ± 0.6	p < 0.001
Phosphorus (pre/post – meq / L)	6.0 ± 0.72 5.3 ± 0.53	p < 0.001
Potassium (pre/post – meq / L)	6.3 ± 0.5 / 5.4 ± 0.48	p < 0.001
Albumin (pre/post – g / dL)	2.9 ± 0.33 / 3.19 ± 0.34	p < 0.001

M: Mean; SD: standard deviation; Kt/V: dialysis treatment adequacy; K: dialyzer clearance of urea; t: dialysis time; V: volume of distribution of urea, approximately equal to patient's total body water; PTH: parathyroid hormone; pre: pre-procedure; post: post-procedure; P: p value

CRI was observed in 41.6% patients (33.3% - CRBSI / 8.3% exit site infection - Table 3) with a mean time of 98 ± 72.1 catheter-days for the first infection (range 6-201). Indications for catheter exchange/removal related to infection did not occur. Seventy-five percent of the bacteria isolated from blood culture were staphylococcal sub-species. In our series, we had an infection rate of 1.67 / catheter days. Because these were patients with vascular access failure, all CRIs were treated with broad spectrum antibiotics, preferably with vancomycin (1000 mg q48hr) and ceftazidime (500 mg q48hr) or amikacin (500mg q48hr) during 14 - 21 days.

**Table 3:** Catheter related infection.

Overall (%)	41.6
Phlebitis (%)	-
Exit site infection (%)	8.3
Pocket infection (%)	-
CRBSI (%)	33.3

CRBSI: catheter related blood stream infection

### Catheter patency

**Figure 2:**
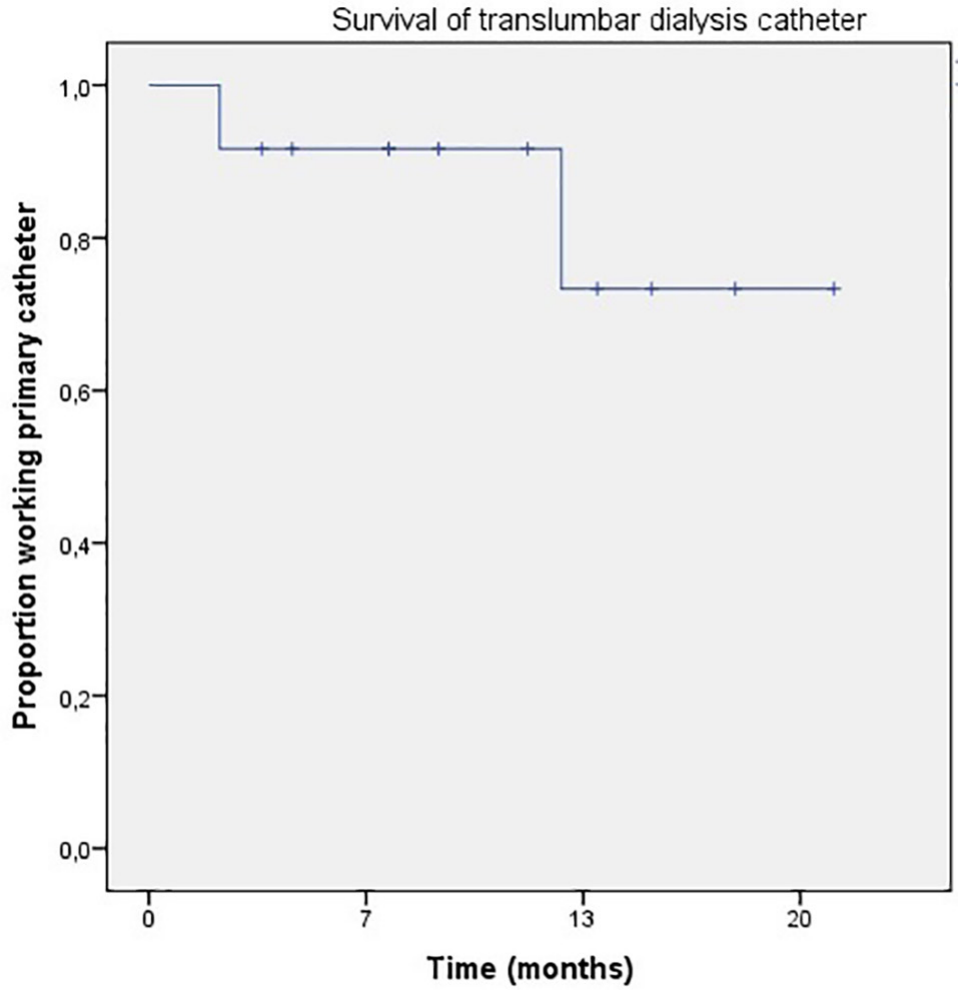
TDLC patency.

Catheters were in place for a total of 3786 catheter days and the average duration of catheters was 315.5 catheter days (range 65 - 631), with only two catheters lost. When censored for elective catheter removal and patient death, the patency was 356 catheter days. One hundred percent of patients in the study retained the initial TLDC beyond 30 days (Figure 2).

### Patient outcomes

Two patients died, both due to vascular access failure (probably due to hyperkalemia and / or hypervolemia), one with 65 catheter days and the other with 380 catheter days.

## DISCUSSION

Among patients who have been on dialysis for a long period, conventional vascular accesses to make an AVF or to implant a catheter might fail, especially in patients with peripheral vascular disease, *multiple previous* access attempts, and multiple comorbidities. Our data suggest that translumbar catheter is an acceptable alternative for these patients. In our series, TLDCs were placed successfully in all patients with good results.

Some studies discourage TLDC use because of low patency. Liu F *et al* 3 observed catheter patency at 3, 6, and 12 months of 43, 25, and 7%. Poor patency rate was attributable to high rate of late thrombosis. In our data catheter patency at 3, 6, and 12 months was 91, 75, and 45%. 

A possible explanation for our better results is the pursuit for appropriate catheter positioning during the procedure, with meticulous detection and correction of kinks and misplacement. Once the catheter is placed, the test of the three Ts is essential: tip, top, and tug^6^. Through use of fluoroscopy, the placement of the *tip* of the catheter should be confirmed, making sure that it does not abut the vessel wall, and the *top* of the catheter should be evaluated to ensure a smooth curve without any kinks. The *tug* test refers to the rapid flow of blood when a 10-mL syringe is attached to both venous and arterial ports and vigorously flushed. Another explanation is the great concern from the dialysis clinic and from the patient; since it may be the last vascular access, hygiene and caution during the handling increases.

Median blood flow rate in our catheters was 300-350 mL/min. According to other authors this is considered an adequate flow for a translumbar device^7^. Lund *et al* 8 defined as translumbar catheter failure a blood flow rate less than 200 mL/min. In this situation, some interventions are necessary to assess eventual thrombus formation and/or improper catheter positioning. Such measures include clot-dissolving medication (urokinase) and venography with catheter repositioning. Catheter removal or replacement must be done only when these measures prove unsuccessful.

Two important limitations of our study are the retrospective nature of the analysis and the small sample of patients. Even so, it was possible to show security in TLDC implant and good patency.

Although the TLDCs should be an alternative until kidney transplantation, only 41.6% of our patients were active in waiting lists for transplantation. Some had absolute contraindications for surgery, like cancer. However, most of them were not on the transplant list due to poor socio-cultural conditions and difficulty to access pre-transplantation exams. This is a harsh reality that needs improving.

In summary, TLDC placement is feasible and relatively safe. It is a salvage way to maintain alive selected patients in HD and it should be used as a bridge to a kidney transplantation. Like traditional CVCs, the most common complications are infection and thrombosis^8^.
